# Application of Twin Screw Extrusion in the Manufacture of Cocrystals, Part I: Four Case Studies

**DOI:** 10.3390/pharmaceutics3030582

**Published:** 2011-08-31

**Authors:** Dominick Daurio, Cesar Medina, Robert Saw, Karthik Nagapudi, Fernando Alvarez-Núñez

**Affiliations:** Small Molecule Process and Product Development, Amgen Inc, Thousand Oaks, CA 91320, USA

**Keywords:** twin screw extrusion, cocrystals, environmentally friendly, green chemistry

## Abstract

The application of twin screw extrusion (TSE) as a scalable and green process for the manufacture of cocrystals was investigated. Four model cocrystal forming systems, Caffeine-Oxalic acid, Nicotinamide-trans cinnamic acid, Carbamazepine-Saccharin, and Theophylline-Citric acid, were selected for the study. The parameters of the extrusion process that influenced cocrystal formation were examined. TSE was found to be an effective method to make cocrystals for all four systems studied. It was demonstrated that temperature and extent of mixing in the extruder were the primary process parameters that influenced extent of conversion to the cocrystal in neat TSE experiments. In addition to neat extrusion, liquid-assisted TSE was also demonstrated for the first time as a viable process for making cocrystals. Notably, the use of catalytic amount of benign solvents led to a lowering of processing temperatures required to form the cocrystal in the extruder. TSE should be considered as an efficient, scalable, and environmentally friendly process for the manufacture of cocrystals with little to no solvent requirements.

## 1. Introduction

Pharmaceutical scientists are currently facing an increasing number of drug molecules with less than ideal properties such as poor solubility and a lack of acceptable physical and chemical stability [1]. Therefore, significant efforts in pharmaceutical sciences have concentrated on finding alternate solid molecular forms and/or formulations that improve these properties. As part of these efforts, cocrystals have been identified as viable solid forms that may improve the pharmaceutical properties of a development candidate in some instances.

Cocrystals have been defined as crystalline materials that are comprised of two or more components that are solids at room temperature (in order to distinguish them from hydrates and solvates) held together by non-covalent forces [2-4]. The distinction between a salt and a cocrystal lies in the fact that there is no proton transfer occurring between the constituents of a cocrystal. There has been an increased interest in cocrystals in the last few years and a number of publications have highlighted the beneficial properties offered by cocrystals. Examples include: (a) several groups have reported solubility and bioavailability enhancements through cocrystallization [5-12], (b) Trask *et al.* have reported that cocrystallization of Caffeine and Theophylline leads to physical stability enhancement [13,14], and (c) cocrystallization to improve the mechanical properties of drugs has also been reported [15,16]. Due to these reported advantages that cocrystals provide, there has been a lot of effort invested in the pharmaceutical industry in identifying, synthesizing, and manufacturing these materials. To this end, there are many reports that detail how to identify and make cocrystals at a small scale [17-22]. However in order to be considered viable candidates for development, a scalable method to produce cocrystals must be established. Traditionally cocrystals have been prepared by slow evaporation or by neat-grinding of the constituents [23,24]. Recently, liquid-assisted grinding has been developed as a more effective method to make cocrystals [25,26]. In addition cocrystals have also been prepared by melt crystallization, sublimation, and solution crystallization. Of these techniques only solution crystallization has proven to be a practical scalable process, due to the ease in reproducibility, phase control, and particle size control. However, the use of solution crystallization for cocrystal scale up does present challenges as knowledge of the ternary phase diagram between the cocrystal constituents and the solvent is necessary and the measurement of such phase diagrams involves a large number of experiments which may be cumbersome to perform [27-29]. In recent years significant progress has been made towards designing simple procedures for using solution crystallization to scale-up the production of cocrystals [30,31]. Nevertheless, use of solution crystallization requires drying of solvent from the final product to acceptable levels and also introduces the risk of forming solvates. We had recently introduced twin-screw extrusion (TSE) of cocrystal components as a scalable and solvent-less process that provides a viable alternative to solution crystallization [32]. Using this process we demonstrated continuous production of cocrystals of Caffeine-Oxalic acid and AMG 517-Sorbic acid. Dhumal *et al.* have also reported the use of extrusion to make Ibuprofen-Nicotinamide cocrystals [33,34]. A detailed study of the effect of temperature and processing conditions on Ibuprofen-Nicotinamide cocrystal formation was reported. They confirmed that extrusion was an excellent method of producing cocrystals while at the same time being easily amenable to a quality-by-design (QbD) approach (which is emerging as a new paradigm in manufacture of pharmaceutical materials). In this paper, we add to understanding and use of TSE in the production of cocrystals. In Part I of this paper we discuss the use of this technique to produce cocrystals of the following systems: Caffeine-oxalic acid, Nicotinamide-Cinnamic acid, Carbamazepine-Saccharin, and Theophylline-Citric acid. All these systems have been extensively studied in literature enabling a comparative analysis between TSE and other methods of forming cocrystals. In a subsequent paper (Part II) we will present a detailed study of AMG517-Sorbic acid cocrystal system comparing TSE and solution crystallization.

## 2. Experimental Section

All chemicals used in the study were purchased from Sigma-Aldrich company (St. Louis, MO, USA) and were used as-received for milling and extrusion experiments. All solvents used were of analytical grade.

### 2.1. Ball Milling

A Retsch Ball Mill model #MM301 was used to grind cocrystal components in order to obtain reference cocrystal material. Where applicable, literature procedures were followed to generate the cocrystals. In general, the samples to be milled were placed in a 25-mL chamber with metal grinding balls at room temperature. The vibration frequency of the mill was set at 15 Hz for all experiments. The samples were milled for varying lengths of time between 0 and 90 minutes. Cocrystals formed after milling were assessed using X-ray powder diffraction and ^13^C solid state NMR to determine the extent of cocrystallization. Milling samples were used as reference material against which cocrystals generated by TSE were compared.

### 2.2. Twin Screw Extrusion (TSE)

**Figure 1.**
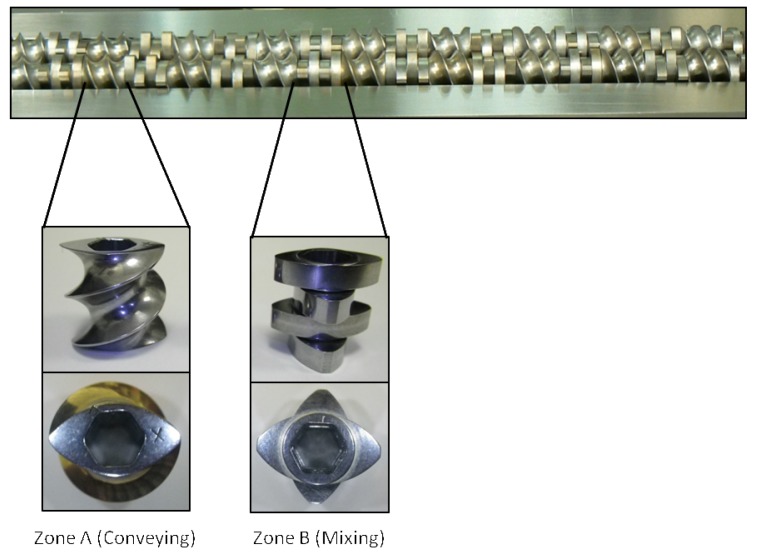
Screw design employed for TSE showing conveying and mixing elements.

TSE experiments were done to determine the feasibility of producing cocrystals in the four aforementioned techniques. A Prism PharmaLab 16mm twin screw extruder (25:1 L/D) was used for all experiments. The extruder has four controllable temperature zones (including the die zone). For these experiments the die zone was not used. Temperature of the extruder barrel was changed according to the model system studied. Appropriate stoichiometric blends of the starting components were prepared and they were charged to extruder manually or through a Brabender single screw volumetric feeder that fed directly into the extruder hopper at a 5% feed rate. The screw was designed for high mixing capacity and long residence time to enhance conversion to cocrystal (cf. Figure 1). The screw design was setup with alternating 16-mm segments of Zone A and Zone B throughout the barrel. Zone A is a purely conveying zone with minimal mixing capacity. Zone A is a single element, 16 mm in length with a pitch of 7.5 mm. Zone B is a highly mixing zone with neutral conveying capacity. Zone B is composed of four distributive elements that are 4 mm in length each for a total of 16 mm. The elements in Zone B are offset by 90° between each element (accomplished by combining alternating 0° and 90° paddle elements). Liquid-assisted extrusion was performed with appropriate amounts of benign liquids added to the powder blends prior to extrusion. All remaining details of the extrusion experiments are provided in the discussion section for each of the systems studied. Each experiment processed anywhere between 20–100 grams of material.

### 2.3. X-Ray Powder Diffraction (XRPD)

The diffractometer (PANalytical X'pert, Philips) was equipped with a CuKα source (λ = 1.54056 Å) operating at a tube load of 45 kV and 40 mA. The divergence slit size was 1/4°, while the receiving slit and the detector slit, were 5.0 mm, and 0.1 mm respectively. A Small amount of sample was loaded onto Si 510 zero-background sample holder and scanned between 3 and 40° (2θ) with a step size of 0.008 and a step time of 15.2 s/step in the continuous mode. Data was collected by a high-resolution sealed proportional detector. The Si (111) with a diffraction peak at 28.44° 2θ was used as a standard to calibrate the instrument.

### 2.4. Differential Scanning Calorimetry (DSC) and Thermo gravimetric Analysis (TGA)

DSC measurements were conducted in crimped Aluminum pans using a Q200 (TA Instruments, NewCastle, DE) unit under 50 mL/min N2 purge. 3 to 5 mg samples were tested each time. Standard DSC scan using a heating rate of 10 °C/min was used. Indium was used as the calibration standard.

TGA measurements were conducted in Aluminum pans using a Q500 (TA Instruments, NewCastle, DE) unit under 50 mL/min N2 purge. A heating rate of 10 °C/min was used for all TGA runs.

### 2.5. Solid State Nuclear Magnetic Resonance (SSNMR)

All SSNMR measurements were conducted using a Bruker DSX spectrometer operating at a ^1^H resonance frequency of 600 MHz. A Bruker 4-mm double resonance magic angle spinning (MAS) probe head was used to record all NMR data. ^13^C NMR spectra were 512 transients were collected for each sample for signal to noise averaging. ^1^H 90° pulse length of 2.5 μs and a cross polarization contact time of 2 ms were employed. ^1^H decoupling was achieved with a spinal 64 sequence using a pulse length of 5 μs. A recycle delay of 10–60 seconds was used depending upon the sample. A total suppression of spinning sidebands (TOSS) sequence was appended to the cross polarization (CP) sequence to achieve a spectrum free of spinning sidebands. Natural abundance Glycine with the carbonyl peak at 176.03 ppm was used as the chemical shift reference.

## 3. Results and Discussion

### 3.1. Caffeine-Oxalic Acid System

**Figure 2.**
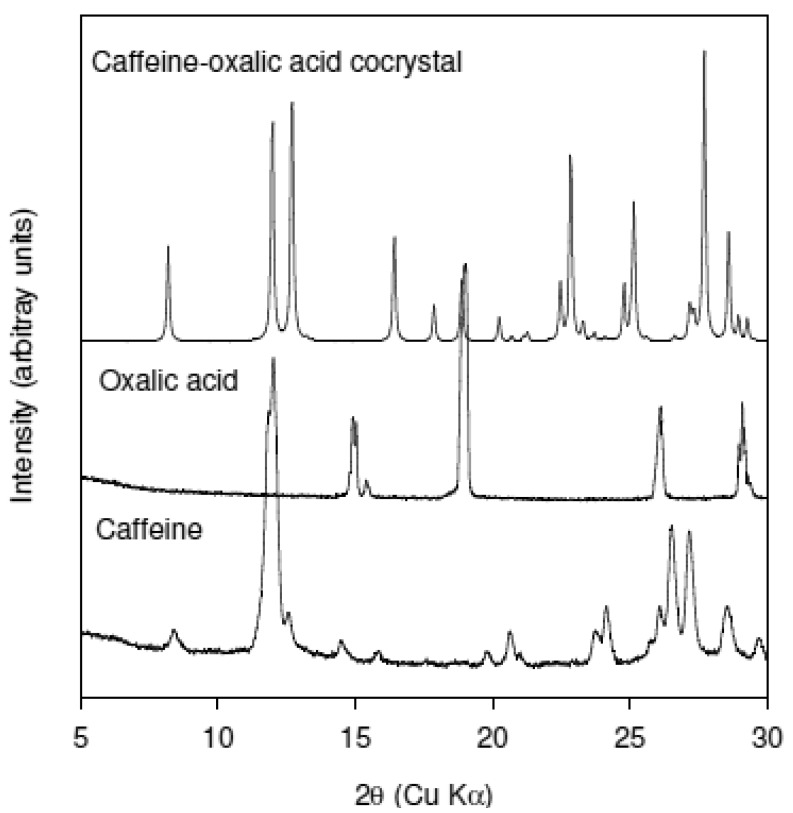
XRPD patterns of as-received Caffeine and as-received Oxalic acid, and Caffeine-oxalic acid cocrystal. Pattern for Caffeine-Oxalic acid cocrystal was calculated from the published crystal structure obtained from the Cambridge Structural Database (CSD).

Trask *et al.* were the first to report a systematic study of Caffeine-based cocrystals [13]. The object of their study was to determine if cocrystal formation could alleviate hydrate formation in Caffeine. They reported the formation of Caffeine-Oxalic acid cocrystal in the stoichiometry of 2:1 using both solution precipitation and solid state grinding methods. Solid state grinding was accomplished using a Retsch mill at a 30-Hz. In addition they also solved the crystal structure of the Caffeine-Oxalic acid cocrystal. Based on the availability of this data set, Caffeine-Oxalic acid system was selected as the model to determine if twin screw extrusion could replicate the results of solid state grinding experiments. Figure 2 shows the XRPD patterns of the as-received caffeine, as-received oxalic acid, and the cocrystal. The XRPD pattern of the cocrystal was simulated from the single crystal data. Closer examination of the XRPD data in the region 9 to 14° 2θ shows that Caffeine has intense reflection at 12° 2θ while the cocrystal has two reflections at 12 and 12.7° 2θ. There is no interference from Oxalic acid in the 9 to 14° 2θ range. This region of the XRPD pattern was selected to monitor conversion to the cocrystal.

The XRPD method used in this paper was intended to provide only a qualitative description of extent of conversion to the cocrystal. No attempt has been made to quantify this method and as such no claims are made regarding the quantitative extent of conversion to the cocrystal. Where possible, cocrystal formation has been confirmed by orthogonal techniques such as ^13^C SSNMR.

In addition to testing the feasibility of cocrystal production using twin screw extrusion, the parameters that could potentially affect the formation of the cocrystal were also investigated. Temperature, extent of mixing, and residence time were identified as the key parameters that could influence cocrystal production. In order to isolate the effect of temperature, a screw design consisting of only conveying elements was employed. Such a design does not provide any mixing in the extruder and as such only the effect of temperature can be studied. Prior to the extrusion experiments Caffeine and Oxalic acid were blended together in the molar ratio of 2:1 and this sample was labeled as the physical blend.

**Figure 3.**
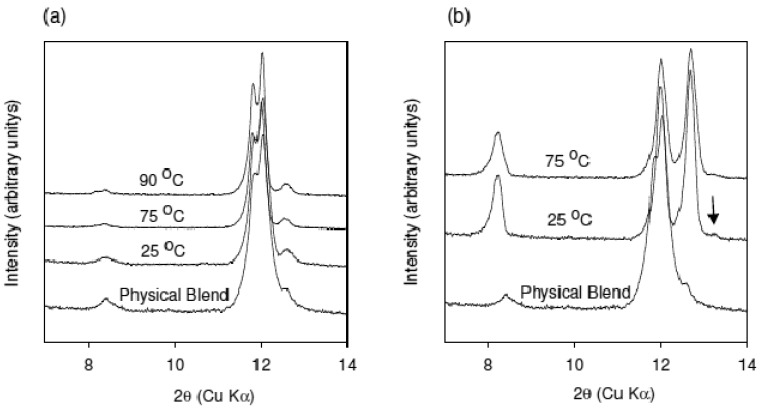
(a) XRPD patterns of the material obtained from using the conveying design at 25, 75, and 90 °C shown in comparison to the physical blend. (b) XRPD patterns of the material obtained from using the mixing design at 25 and 75 °C shown in comparison to the physical blend. New reflection at 13.2° 2θ is marked with an arrow. These data show that mixing in the extruder is essential for cocrystal formation.

XRPD patterns of the material obtained from using the conveying design at 25, 75, and 90 °C are shown in comparison to the physical blend in Figure 3(a). The temperatures used for the experiment were below the melting point of either caffeine or oxalic acid. It is clear from the XRPD patterns that no conversion to the cocrystal has occurred at any of the applied temperatures. These data show that the cocrystal formation is not mediated by temperature in the extruder under the given experimental conditions. Moreover, these data suggest that eutectic formation may not be the mechanism controlling cocrystal formation in the caffeine-oxalic acid case.

**Figure 4.**
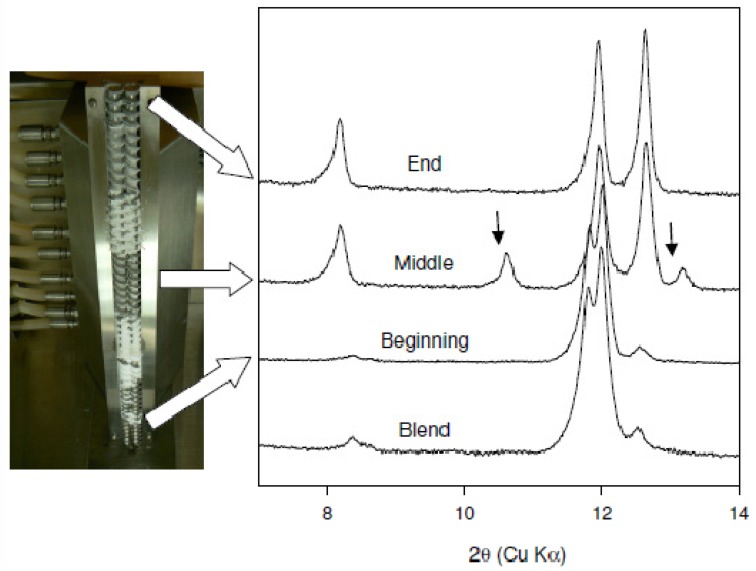
XRPD patterns showing the progressive conversion to the cocrystal for samples removed from the beginning, middle and end of the extruder barrel. New reflections at 10.6 and 13.2° 2θ are marked with an arrow.

The effect of mixing on cocrystal formation was examined by conducting extrusion experiments at 25 °C and 75 °C with a customized screw design employing several mixing elements. XRPD data of the materials obtained from these experiments are shown in comparison to the preblend in Figure 3(b). Conversion to the cocrystal was observed at both temperatures. However, two additional reflections at 10.6 and 13.2° 2θ are observed for the sample extruded at 25 °C (marked with an arrow in the figure). These reflections have also been observed in other samples prepared at different temperature conditions. As an example, XRPD of the material recovered from the beginning, middle, and end of the extruder barrel for the experiment run at 75 °C is shown in Figure 4. The new reflections at 10.6 and 13.2° 2θ are more prominent in the sample removed from the middle of the barrel. These reflections are not present in the sample removed from the end of the barrel. In addition, these reflections were also found to disappear over time regardless of the processing conditions. Based on this observation, the new reflections are thought to be arising from a metastable polymorph of the caffeine-oxalic cocrystal.

This mixing experiment clearly shows that the customized screw design provides very efficient mixing in the extruder barrel and continuously creates new surface contacts promoting cocrystal formation. As the cocrystal formation occurs at both 25 °C and 75 °C when the mixing screw design is used, it further indicates that mechanism may not be mediated by a liquid phase formation due to the eutectic temperature.

**Figure 5.**
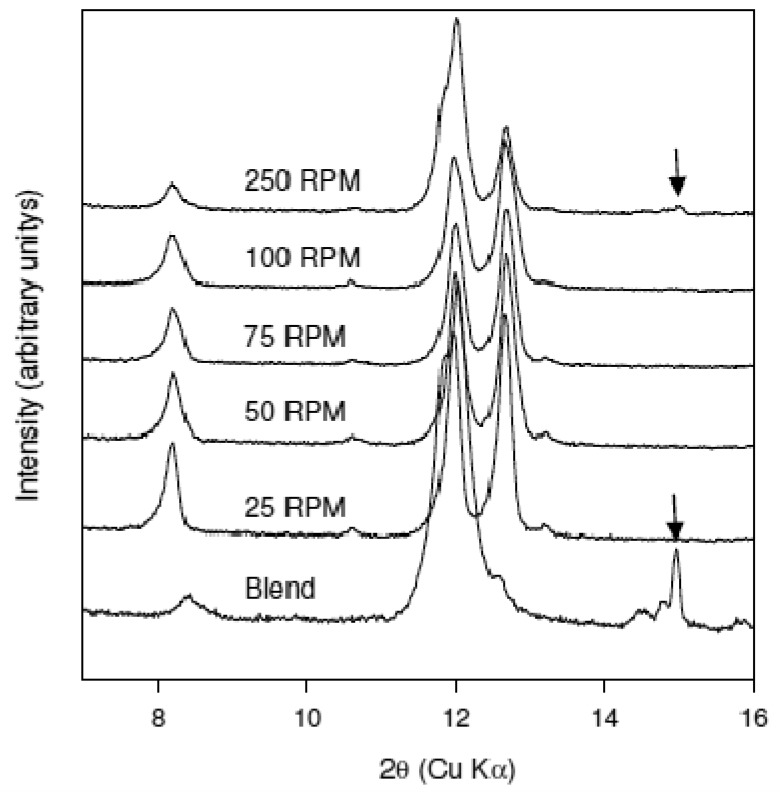
XRPD patterns of the material obtained from using the mixing design at 25 °C as a function of screw speed shown comparison to the preblend. Caffeine reflections are marked with an arrow. Screw speed (residence time) has a minimal impact on the extent of conversion to the cocrystal.

The effect of residence time on cocrystal production was examined at 75 °C by continuously varying screw speed from 25 to 250 RPM. It is assumed that the lower the RPM the larger is the residence time in the extruder. XRPD data of the materials obtained from these experiments are shown in comparison to the physical blend in Figure 5. The data indicate that conversion to the cocrystal has occurred at all screw speeds. However, for material processed at the 250 RPM screw speed residual caffeine reflections were detected (marked with an arrow in Figure 4) indicating incomplete conversion to the cocrystal. Thus, as anticipated, employing short residence times (high screw speeds) leads to a decrease in the extent of conversion to the cocrystal. Nevertheless, the dependence of the extent of conversion to the cocrystal on the screw speed is weak since only the highest screw speed gave incomplete conversion.

**Figure 6.**
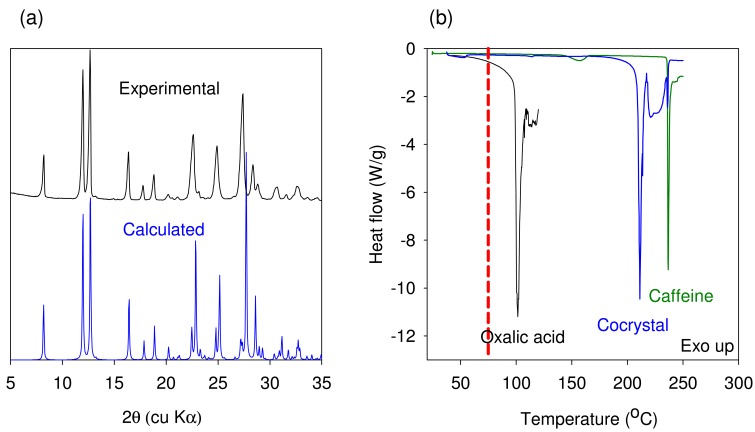
(a) Comparison of experimental and calculated XRPD profiles of caffeine-oxalic acid cocrystal. (b) DSC thermogram showing comparison of oxalic acid dihydrate, anhydrous caffeine, and 2:1 caffeine-oxalic acid cocrystal. The dotted line in Figure (b) represents the extrusion temperature.

Finally a scale up of cocrystal production (100 gram scale), was attempted using the mixing screw design at 75 °C at a 5% feed rate and 100 RPM screw speed. Figure 4 shows the progressive conversion to the cocrystal through the length of the barrel. The experimental and predicted powder X-ray diffraction patterns of caffeine-oxalic acid cocrystal are shown in Figure 6(a). XRPD pattern of the cocrystal manufactured using the TSE process was found to be in agreement with the calculated powder pattern corresponding to the published crystal structure obtained from the Cambridge Structural Database (CSD). The DSC thermograms of caffeine, oxalic acid, and the cocrystal are shown in Figure 6(b). The peak melting temperature of the cocrystal was found to be in-between that of the starting components. Thus, thermal data provide further confirmation of the cocrystal formation by the TSE process. Based on these data, twin screw extrusion was found to be a viable option for producing Caffeine-oxalic acid cocrystals.

### 3.2. Nicotinamide-trans Cinnamic Acid System

Nicotinamide- *trans* cinnamic acid cocrystal system has been extensively studied. Quehenberger has reported the binary phase diagram between *trans* cinnamic acid and Nicotinamide [23]. Two different polymorphic modifications for the Nicotinamide-*trans* Cinnamic acid 1:1 cocrystal system with melts at 98.5 °C and 96.5 °C were reported. In a more recent report Chiarella and co-workers have described the ternary phase diagram of the Nicotinamide and *trans* Cinnanmic acid in methanol at 20 °C and in water at 50 °C [27]. Their work showed that when starting with a 1:1 mixture of the pure components, cocrystals are produced as the equilibrium phase in methanol and not in water. They were able to rationalize the results using the ternary phase diagram. This example illustrates that even though solution crystallizations can be developed for cocrystal production it does involve numerous careful and tedious experiments to develop the process. In contrast, melt crystallization of the binary system consisting of the pure components was found to yield the cocrystal in a straightforward manner. Thus, Nicotinamide-*trans* Cinnamic acid system was used as the second model system to illustrate the use of TSE in continuous production of cocrystals.

**Figure 7.**
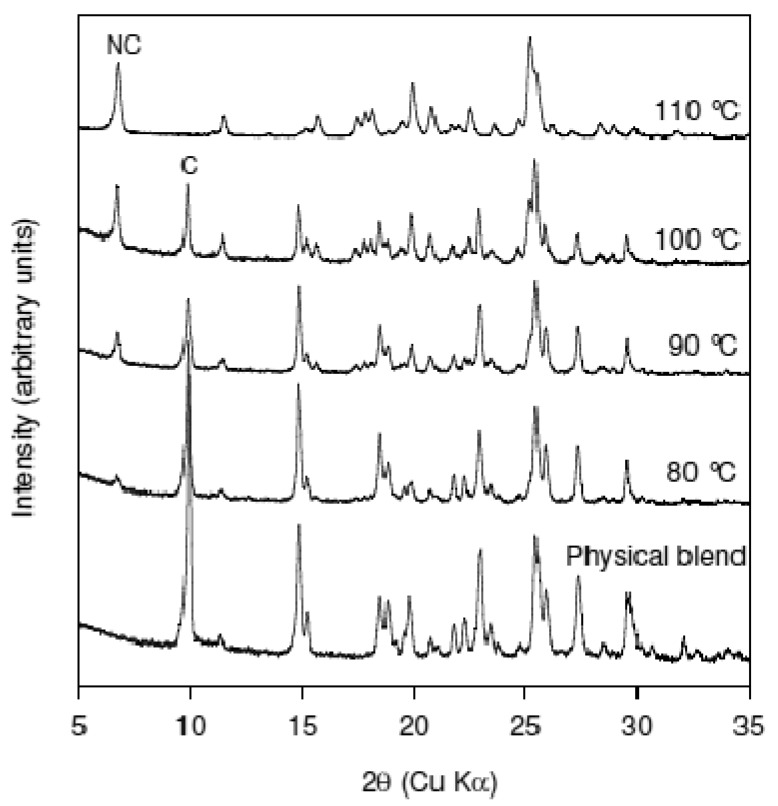
XRPD patterns of materials produced from extrusion of Nicotinamide-trans cinnamic acid blend at 80, 90, 100, and 110 °C at 75 RPM screw speed. The peak corresponding to Cinnamic acid is labeled ‘C’ while the peak corresponding to the cocrystal is labeled as “NC”. The data shows that extent of cocrystal formation is the highest at 110 °C.

The effect of temperature on cocrystal formation was investigated using manual feeding to the extruder at 75 RPM. Figure 7 shows the XRPD patterns of materials produced from extrusion of Nicotinamide-trans cinnamic acid blend at 80 °C, 90 °C, 100 °C, and 110 °C. The peak corresponding to Cinnamic acid is labeled ‘C’ while the peak corresponding to the cocrystal is labeled as “NC”. The XRPD patterns show the progressive conversion to the cocrystal from the physical blend. At the experimental conditions used, it was determined that 110 °C was required to achieve maximum cocrystal conversion. The sample recovered from the extruder for experiments done at 120 °C also showed good conversion to the cocrystal (data not shown). However, at 120 °C power over-torque and clumping in the extruder was observed which precluded the use of this temperature to generate the cocrystal. In contrast to the Caffeine-Oxalic acid cocrystal system, the extent of conversion to the cocrystal in the Nicotinamide-trans Cinnamic acid system was found to be temperature dependent, at least in the experimental conditions studied. These data show that eutectic formation is involved in cocrystal formation for this system (as has been previously published).

**Figure 8.**
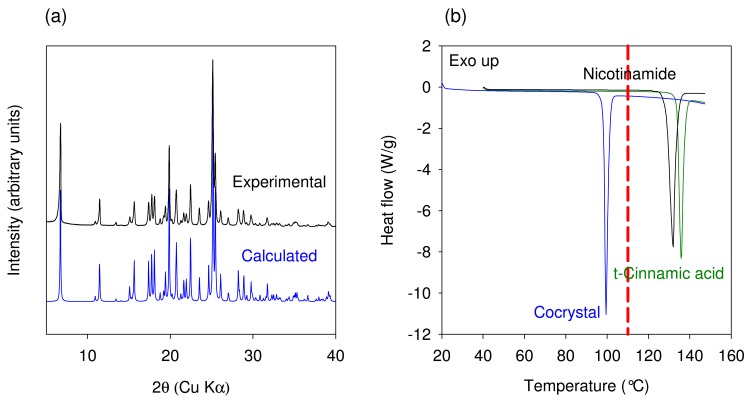
(**a**) Comparison of experimental and calculated XRPD profiles of Nicotinamide-trans Cinnamic acid cocrystal. (**b**) DSC thermogram showing comparison of trans cinnamic acid, Nicotinamide and the 1:1 Nicotinamide-trans Cinnamic acid cocrystal. The dotted line in Figure (b) represents the extrusion temperature.

The experimental and predicted powder X-ray diffraction patterns of Nicotinamide-trans Cinnamic acid cocrystal are shown in Figure 8(a). The XRPD pattern of the cocrystal manufactured using the TSE process was found to be in agreement with the calculated powder pattern corresponding to the crystal structure obtained by Chiarella *et al.* The DSC thermograms of Nicotinamide, Cinnamic acid, and the cocrystal are shown in Figure 8(b). The melting temperature onset (98.4 °C) of the cocrystal was found to be lower than that of the starting components. Thus, thermal data provide further confirmation of the cocrystal formation by the TSE process. The XRPD pattern and melting temperature compare well with published data for Form I of the cocrystal. The data generated using this model system illustrates that the temperature in the extruder for maximizing cocrystal conversion needs to be set depending upon the observed results from the extruder. However, a better approach would involve generating binary phase diagrams using DSC (approach involves minimal use of material) to understand if cocrystal formation is indeed mediated by eutectic formation. For such systems, the extrusion must be carried out at temperatures greater than the eutectic temperature.

### 3.3. Carbamazepine-Saccharin System

CarbamazepineCocrystals made from Carbamazepine and Saccharin have been extensively investigated. Nair *et al.* have investigated the formation of this cocrystal using grinding methods [35]. They observed that cocrystal formation was mediated by generation of the amorphous phase. The extent of amorphous formation was found to be dependent on grinding temperature with higher levels of amorphous phase being produced upon cryogrinding. The amorphous phase was found to continuously crystallize on storage into the anhydrous cocrystal Form I. The extent of cocrystal formation from the amorphous phase at room temperature was found to be impacted by relative humidity upon storage. The extent of cocrystal formation was also found to be dependent on the hydration state of the starting material. For example if Carbamazepine dihydrate was used instead of anhydrous Carbamazepine, the rate of cocrystal formation was found to be accelerated for grinding conducted under similar conditions. This result was rationalized by the fact that water in starting material serves as a plasticizer aiding in decreasing the glass transition temperature of the amorphous phase, thereby giving it a better chance to crystallize during grinding. This system was selected as our third model cocrystal to determine if TSE can produce cocrystals where the mechanism of formation is mediated by the amorphous phase.

**Figure 9.**
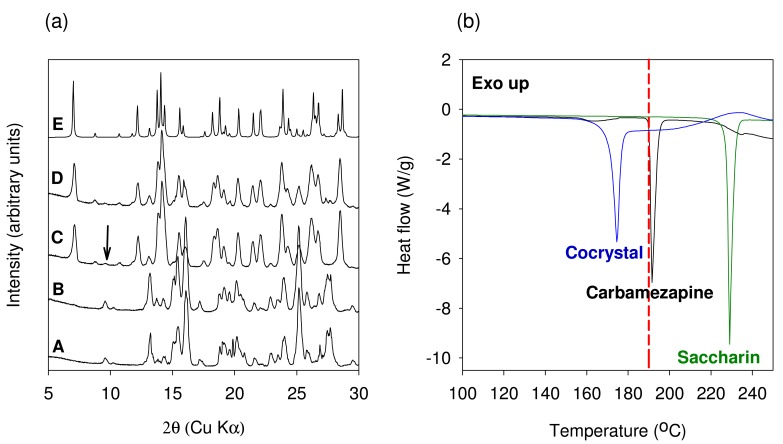
(**a**) XRPD of materials obtained from the TSE of Carbamazepine and Saccharin: A: Physical blend prior to extrusion, B: extrusion at 50 °C, C: Extrusion at 190 °C, D: Extrusion with water at 100 °C, E: Reference Form I cocrystal pattern calculated from the published crystal structure obtained from the Cambridge Structural Database (CSD). (**b**) DSC thermogram showing comparison of Carbamazepine, Saccharin and the 1:1 Carbamazepine-Saccharin cocrystal. The dotted line in Figure (b) represents the extrusion temperature.

The XRPD patterns of the material generated using TSE of anhydrous Carbamazepine and Saccharin are shown in Figure 9(a). No conversion to the cocrystal was observed when the extrusion was conducted at 50 °C with a screw speed of 25 RPM (XRPD pattern B). However, cocrystal formation was observed when the anhydrous materials were co-ground for 30 min in a ball mill. The final bulk temperature after the milling process was found to be 45 °C. Thus the extrusion result does not match the milling result using similar temperature conditions. These results may be explained considering the milling intensity, milling time, and the amount of material processed. Ball milling is a much more energy intensive process than TSE. Ball milling can therefore lead to a greater disruption of the crystalline lattice as compared to extrusion. It was also demonstrated by Nair *et al.* that even with these levels of intensive energy generated by the ball mall, at least 30 minutes of milling time is required to achieve a cocrystal conversion of about 90%. In contrast, the processing time (residence time) in the extruder is in the order of about 2 to 5 min. This value cannot be significantly altered due to practical considerations of extrusion processing. The amount of material processed in the extruder was another point of difference as compared to ball milling. The total amount processed at any given time is about 20 times higher in an extruder than that of a ball mill. Again owing to practical considerations the amount that is processed cannot be reduced below 10 grams in the extruder. Based on this discussion it is clear that co-grinding results using ball milling of anhydrous starting components is not representative of the conditions used in extrusion and should not be expected to yield the cocrystal.

In order to improve cocrystal formation using TSE, the temperature in the extruder was altered. A temperature of 190 °C which is close to the onset of melting of Carbamazepine was selected. XRPD pattern of the material obtained after TSE at 190 °C shows near complete conversion to the cocrystal (XRPD pattern C). Approximately 95% conversion to the cocrystal was calculated using ^13^C SSNMR spectroscopy (Data not shown). Consistent with the SSNMR data XRPD pattern also shows the presence of unreacted material (marked with an arrow in Figure 9(a)). In addition to increasing the temperature further optimization of the extrusion parameters can achieve better conversions to the cocrystal.

An evaluation was also conducted to see if TSE could replicate the published results which show the rate of cocrystal formation increased by the presence of water. In previous reported studies, Carbamazepine dihydrate was used as the starting material and the water in the dihydrate was found to increase the cocrystal conversion [35]. This can be considered as an equivalent of liquid-assisted grinding where liquid is intentionally added to the system to facilitate cocrystal formation. Jones *et al.* have extensively reviewed liquid-assisted grinding [36]. Liquid-assisted grinding was found to be superior to neat grinding in most cases in effecting cocrystal transformation. The liquid is typically used in small amounts and could end up in the final product resulting in the formation of a solvated phase. In the case of anhydrous Carbamazepine-Saccharin cocrystal, the liquid is expected to have only a catalytic role as the cocrystal is anhydrous.

In our studies, we decided to investigate liquid-assisted extrusion by processing the anhydrous starting components in the presence of water. About 5 mL of water was added to 10.4 grams of a 1:1 molar blend of anhydrous Carbamazepine and Saccharin and the extrusion was carried out at 100 °C. Water was chosen as the model liquid phase since use of water does not require any additional environmental controls and 100 °C temperature was chosen to obtain a product free of water. The XRPD pattern of the material obtained from this experiment shows that while conversion to the cocrystal has occurred the degree of conversion is less than what was obtained with neat extrusion (XRPD pattern D). Approximately 87% conversion to the cocrystal was calculated using ^13^C SSNMR spectroscopy (Data not shown). Since this experiment was only intended to demonstrate proof of concept, it was not further optimized. It is believed that with further optimization using a gradient temperature in the barrel better conversions will be observed. The use of hydrated starting material could also be used as a means of improving the extent of cocrystal formation. The DSC thermograms of Carbamazepine, Saccharin and the 1:1 Carbamazepine-Saccharin cocrystal are shown in Figure 9(b). The melting temperature onset (171.6 °C) of the cocrystal was found to be lower than that of the starting components and in good agreement with published data.

Matzger *et al.* have reported the formation of another anhydrous polymorph (Form II) of Carbamazepine and Saccharin [37]. This polymorph was prepared from an ethanol solution in the presence poly(4-methyl 1-pentene) as heteronuclei. TSE was attempted to produce anhydrous Form II by mixing Carbamazepine and Saccharin with ethanol and poly(4-methyl 1-pentene). The temperature along the barrel for this experiment was increased from 20 °C to 80 °C. The final temperature was selected to purge all the ethanol from the system. The experiment resulted in the production of Form I cocrystal (data not shown). While Form II polymorph could not be produced, this experiment showed that either water or ethanol could be used in the liquid-assisted extrusion process to make the Form I cocrystal. Notably, the use of catalytic amounts of benign solvents led to a lowering of experimental temperatures required to form the cocrystal in the extruder.

### 3.4. Theophylline-Citric Acid System

Theophylline and Citric acid are known to cocrystallize in both the anhydrous and hydrated forms (In the hydrated form all three components are present in equal stoichiometric proportions). Jones *et al.* have reported a systematic study of the system and the outcome of their work is summarized in Table 1 [36]. Briefly, the anhydrous cocrystal is formed when neat grinding of the anhydrous reactants is carried out at room temperature. However, the hydrated cocrystal results when either reactant is a hydrate or when both reactants are hydrates. The hydrated cocrystal could also be produced when catalytic amounts of water are added to the system. In the case of liquid-assisted grinding with water, the nature of the starting materials becomes immaterial as the externally added water acts to produce the hydrated cocrystal. In contrast to the previous example, for the Theophylline-Citric acid case, the liquid component ends up in the final cocrystal product. Thus, Theophylline-Citric acid was selected as the final model system to examine if TSE is capable of making both anhydrous and hydrated variety of the cocrystal.

**Figure 10.**
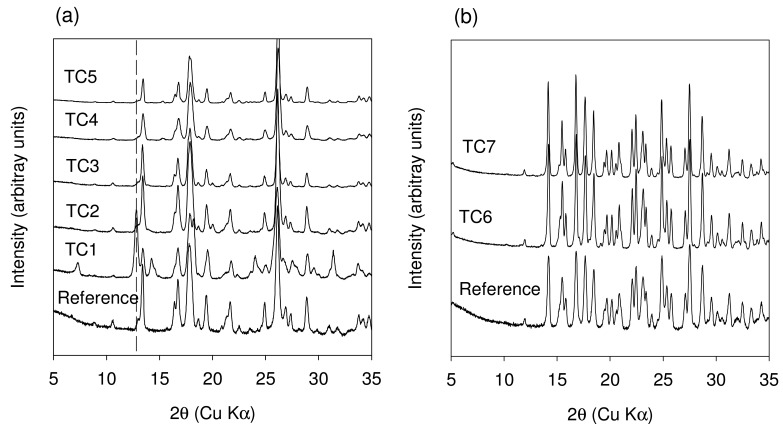
XRPD of materials obtained from the extrusion of Theophylline and citric acid. The descriptions of samples TC1 through TC7 are provided in Table 2. (**a**) Samples producing the anhydrous cocrystal phase; and (**b**) samples producing the hydrated cocrystal. The dotted line in Figure (a) tracks the peak at 12.8° 2θ and provides information about unreacted starting material in the extruded samples.

The list of TSE experiments conducted with this system along with the outcome of the experiments is summarized in Table 2. The outcome of the TSE experiments mirrored the findings observed with the grinding experiments. However, TSE experiments differ in one important aspect as compared to grinding. The difference is that a wider range of controlled temperatures can be used with TSE experiments. As with the grinding experiments, hydrated cocrystal was produced in TSE at room temperature when the starting reactant contained water. As an example, the TSE at room temperature of a blend of anhydrous Theophylline and citric acid monohydrate (sample TC6 in Table 2) produced the hydrated cocrystal. Hydrated cocrystal was also produced when the sample blend was subjected to TSE with a catalytic amount of water added to it (sample TC7 in Table 2). The XRPD patterns of TC6 and TC7 are shown in comparison with a reference hydrated cocrystal pattern in Figure 10(b). In contrast to extrusion at 20 °C, extrusion of a blend of anhydrous Theophylline and citric acid monohydrate at 50 °C produced the anhydrous cocrystal (sample TC4 in Table 2). Thus by simply modifying the extrusion temperature, the same blend of starting materials can be made to produce the anhydrous cocrystal.

When the extrusion temperature was increased to 153 °C, as expected, the anhydrous cocrystal was formed (sample TC2 in Table 2). Regardless of the starting components (samples TC1, TC2, and TC3 in Table 2) when the extrusion was carried out at 153 °C the anhydrous cocrystal was produced. The XRPD patterns of the materials TC1 through TC4 are shown in comparison to the reference anhydrous cocrystal in Figure 10(a). Following the reflection at 12.8° 2θ (characteristic of anhydrous Theophylline) it is clear that samples TC1 through TC3 have unreacted starting components. Only sample TC4 was found to be converted almost entirely to the cocrystal. In addition the extent of conversion to the anhydrous cocrystal was found to be higher for TC2 and TC3 when compared to TC1.

**Figure 11.**
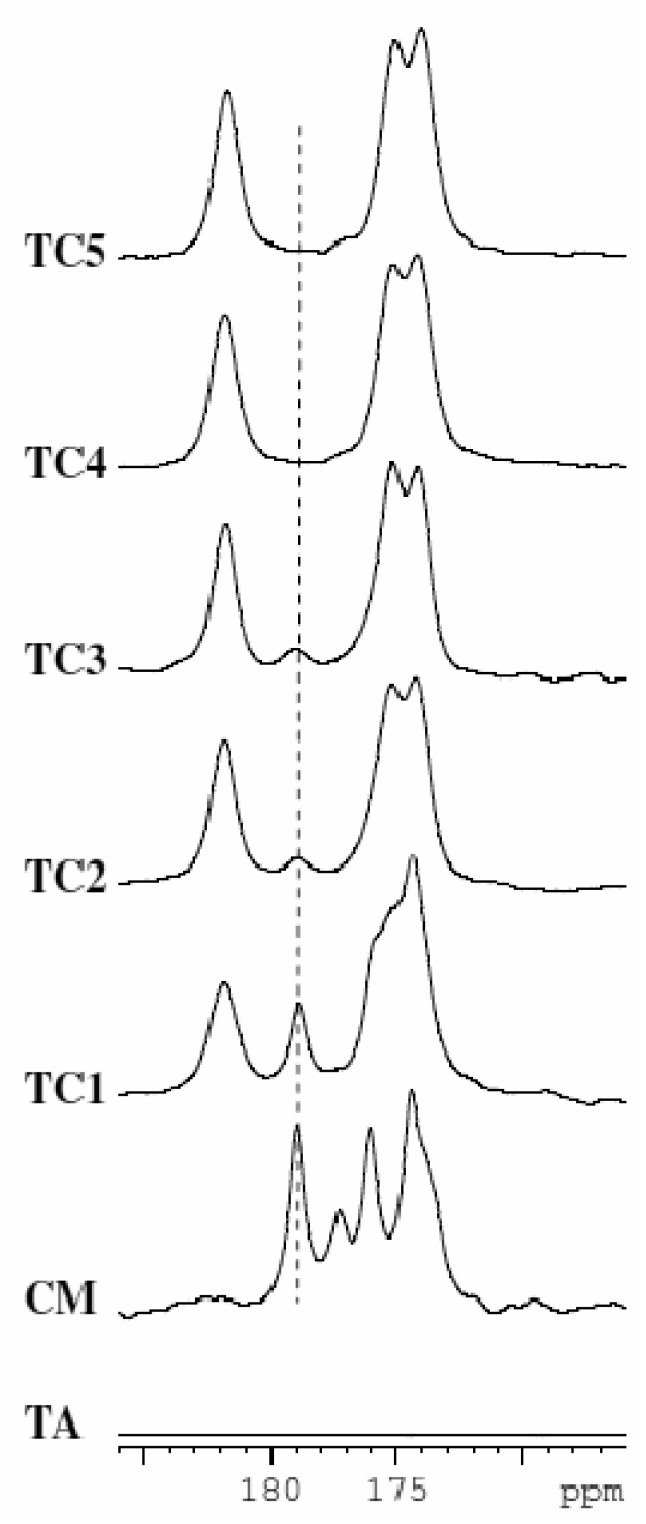
^13^C CP/MAS spectra of samples TA: Anhydrous Theophylline (has no peaks in this ppm range), CM: Citric acid monohydrate, TC1, TC2, TC3, TC4, and TC5 (sample descriptions are shown in Table 2). The extent of conversion to the cocrystal follows the order TC1 < TC3 < TC2 < TC4 = TC5.

Thus, when extrusion is carried at 20 °C as in the case of TC6, water resides in the crystal lattice in the form of a hydrated cocrystal. However when extrusion is carried out at 50 °C as in the case of TC4, water serves to aid in the cocrystal formation of the anhydrous phase. Even when extrusion is carried out at 153 °C as in the case of TC2 and TC3 water aids in conversion to the anhydrous albeit not completely. However in the absence of water, when extrusion is carried out at 153 °C as in the case of TC1, the extent of conversion to the cocrystal was found to be the lowest. These observations point to the fact that presence of a liquid phase (water in this case which comes through the starting reactants) aids in formation of the anhydrous and hydrated cocrystal. In order to further show the utility of liquid-assisted extrusion, TSE of a blend of anhydrous Theophylline and anhydrous Citric acid was carried out at 20 °C in the presence of ethanol (sample TC5 in Table 2). In this case complete conversion to the cocrystal was observed. ^13^C solid state NMR data in the region of the carboxyl group is shown for materials TC1, TC2, TC4, and TC5 in comparison to the starting materials in Figure 11.

The peak at 178.9 ppm corresponding to citric acid monohydrate was followed to determine extent of conversion to the cocrystal. These data clearly show that conversion scales as TC1 < TC3 < TC2 < TC4 = TC5. Thus, as seen in the previous case study with Carbamazepine and Saccharin, liquid-assisted extrusion is able to facilitate cocrystal formation at lower temperatures and results in increased extent of conversion when compared to neat extrusion. These findings using TSE nicely parallel those observed with grinding experiments.

## 4. Conclusions

The application of TSE in the continuous production of cocrystals has been demonstrated for four model cocrystal-forming systems. Extrusion was found to be an effective method to make cocrystals, whether or not the mechanism of formation involved eutectic formation. The presence of mixing and temperature were found to be the critical parameters that influence cocrystal formation during extrusion processing. TSE provides highly efficient mixing and close material packing of components which in turn lead to improved surface contact between components thereby facilitating cocrystal formation without the use of solvents. For the first time, liquid-assisted extrusion has also been demonstrated. The addition of small amounts of benign liquids adds another processing dimension to the extrusion process thereby allowing for further flexibility in optimizing cocrystal production using TSE. Moreover, liquid-assisted extrusion can promote cocrystal formation at lower temperatures obviating the need of high temperature processing. Unlike other mechanical mixing procedures, TSE is a continuous process and lends itself to practical scalability. As shown, extrusion can be considered an efficient, scalable, and environmentally friendly process for the manufacture of cocrystals which provides a viable alternative to solution crystallization processes.

## Figures and Tables

**Table 1.** Outcome of grinding experiments performed with Theophylline-Citric acid system. The data has been summarized from the work of Jones *et al.* [36].

**Component 1**	**Component 2**	**Type of grinding**	**Product**
TA	CA	Neat	AC
TA	CM	Neat	HC
TM	CA	Neat	HC
TM	CM	Neat	HC
TA, TM	CA, CM	Water-assisted	HC

TA: Theophylline Anhydrous; TM: Theophylline monohydrate; CA: Citric acid Anhydrous, CM: Citric acid monohydrate; AC: Anhydrous cocrystal, HC: Hydrated cocrystal.

**Table 2.** Outcome of TSE experiments performed with Theophylline-Citric acid system.

**Lot#**	**Component 1**	**Component 2**	**Type of Extrusion**	**Temperature**	**Product**
TC1	TA	CA	neat	153	AC + unreacted*
TC2	TA	CM	neat	153	AC + unreacted
TC3	TM	CA	neat	153	AC + unreacted
TC4	TA	CM	neat	50	AC
TC5	TA	CA	Ethanol-assisted	20	AC
TC6	TA	CM	neat	20	HC
TC7	TA	CM	Water-assisted	20	HC

TA: Theophylline Anhydrous, TM: Theophylline monohydrate; CA: Citric acid Anhydrous, CM: Citric acid monohydrate; AC: Anhydrous cocrystal, HC: Hydrated cocrystal; * unreacted starting material was observed by XRPD.
